# Supplementary material for: An Injury-like Signature of the Extracellular Glioma Metabolome
Source: Cancers (Basel). 2024 Jul 30;16(15):2705. doi: 10.3390/cancers16152705 (PMC11311774; doi:10.3390/cancers16152705)
Supplement: Supplementary file 1 [file cancers-16-02705-s001.zip › supplementary figures.pdf]

S1A. Enrichment analysis: 72 vs baseline vehicle-treated brain ranked list ("injury signature")

Baseline GBM164 (vs. brain) | **Baseline brain (vs GBM164)**

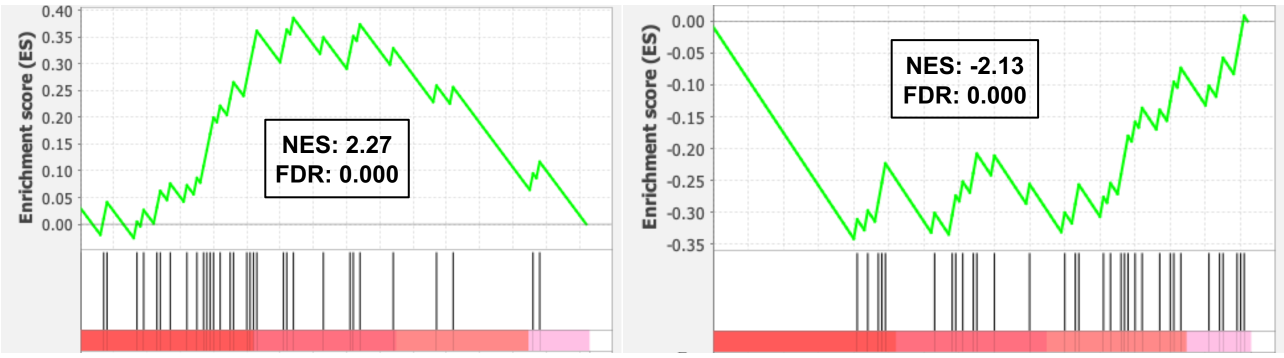

S1B. Enrichment analysis: 72h vs baseline vehicle-treated GBM164 ranked list

Baseline GBM164 (vs brain) | **Baseline brain (vs GBM164)**

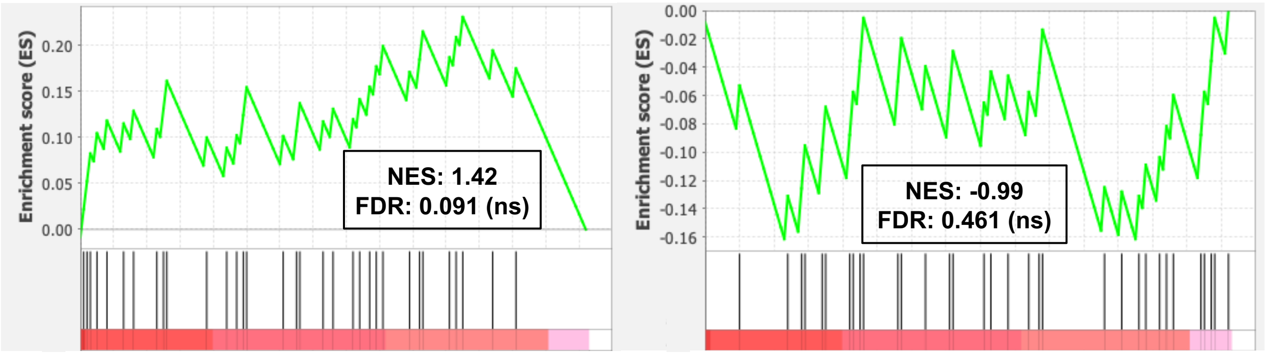

Supplementary Figure S1: S1A. Enrichment plots of the 72-hour vs. baseline of vehicle in brain ranked list with the top and bottom 35 metabolites of average tumor vs. brain signature. S1B. Enrichment plots of the 72-hour vs. baseline of vehicle in tumor ranked list with the top and bottom 35 metabolites of average tumor vs. brain signature.

S2. Enrichment analysis: High-plasma versus low-plasma human CSF

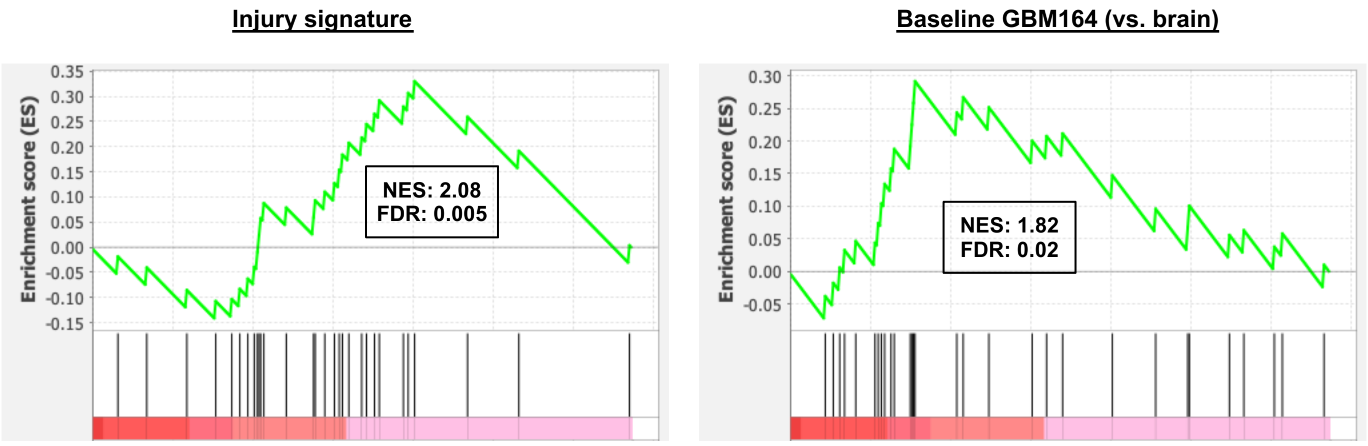

Supplementary Figure S2: Enrichment plots of the high-plasma vs. low-plasma human CSF ranked list with the top 35 metabolites of injury signature and murine tumor signature.
